# Obituary

**Published:** 1894-08

**Authors:** 


OBITUARY.—Dr. Chas. Antis Danforth, of Granger, Texas, died at his home on Sunday, May 6, 1894. He was an active member of the Williamson-Bell-Milam-County Medical Society, and the Society passed resolutions expressive of their sense of loss in so valuable a member, and testifying to his high character and general worth as a physician and a citizen. A beautifully-written eulogy on his life and character was forwarded by the W.-B.-M.-County Medical Society to the TEXAS MEDICAL JOURNAL for publication, but was not published because of its length. From it, however, we make the following extracts:

Dr. Danforth was born at Marshall, Texas, September 18, 1853. He was married to Miss Henrietta G. Gerdes, of Rockdale, Texas, February 6, 1879, and she, with six children, survives him. He chose medicine as a profession, and in 1881 attended his first course of lectures at Missouri Medical College, St. Louis, and began practice at Granger, Texas. He took his second course at Tulane University, New Orleans, in the winter of 1883, and graduated the following spring, March, 1884. Returning to Granger, he located permanently, and there resided and followed his profession with zeal, energy and success, till cut off by an untimely death.

In 1887 Dr. Danforth took a course at the St. Louis Polyclinic, and again, at New Orleans, he attended the Polyclinic in 1889–'90–'91–'93. With these exceptions he was continually in practice, and worked with an ambition worthy of emulation. He was a close student, and his early death may, in a measure, be attributed to his excessive application to work and study. Dr. Danforth was highly respected by all who knew him, and had he lived would undoubtedly have made a brilliant career.

